# AI-AIF: artificial intelligence-based arterial input function for quantitative stress perfusion cardiac magnetic resonance

**DOI:** 10.1093/ehjdh/ztac074

**Published:** 2022-12-07

**Authors:** Cian M Scannell, Ebraham Alskaf, Noor Sharrack, Reza Razavi, Sebastien Ourselin, Alistair A Young, Sven Plein, Amedeo Chiribiri

**Affiliations:** School of Biomedical Engineering & Imaging Sciences, King’s College London, 4th Floor Lambeth Wing, St Thomas′ Hospital, London SE1 7EH, UK; Department of Biomedical Engineering, Eindhoven University of Technology, Gemini-Zuid, Groene Loper 5, 5612 Eindhoven, The Netherlands; School of Biomedical Engineering & Imaging Sciences, King’s College London, 4th Floor Lambeth Wing, St Thomas′ Hospital, London SE1 7EH, UK; Department of Biomedical Imaging Science, Leeds Institute of Cardiovascular and Metabolic Medicine, University of Leeds, Clarendon Way, Leeds LS2 9JT, UK; School of Biomedical Engineering & Imaging Sciences, King’s College London, 4th Floor Lambeth Wing, St Thomas′ Hospital, London SE1 7EH, UK; School of Biomedical Engineering & Imaging Sciences, King’s College London, 4th Floor Lambeth Wing, St Thomas′ Hospital, London SE1 7EH, UK; School of Biomedical Engineering & Imaging Sciences, King’s College London, 4th Floor Lambeth Wing, St Thomas′ Hospital, London SE1 7EH, UK; School of Biomedical Engineering & Imaging Sciences, King’s College London, 4th Floor Lambeth Wing, St Thomas′ Hospital, London SE1 7EH, UK; Department of Biomedical Imaging Science, Leeds Institute of Cardiovascular and Metabolic Medicine, University of Leeds, Clarendon Way, Leeds LS2 9JT, UK; School of Biomedical Engineering & Imaging Sciences, King’s College London, 4th Floor Lambeth Wing, St Thomas′ Hospital, London SE1 7EH, UK

**Keywords:** Artificial intelligence, Arterial input function, Quantitative myocardial perfusion, Cardiac magnetic resonance

## Abstract

**Aims:**

One of the major challenges in the quantification of myocardial blood flow (MBF) from stress perfusion cardiac magnetic resonance (CMR) is the estimation of the arterial input function (AIF). This is due to the non-linear relationship between the concentration of gadolinium and the MR signal, which leads to signal saturation. In this work, we show that a deep learning model can be trained to predict the unsaturated AIF from standard images, using the reference dual-sequence acquisition AIFs (DS-AIFs) for training.

**Methods and results:**

A 1D U-Net was trained, to take the saturated AIF from the standard images as input and predict the unsaturated AIF, using the data from 201 patients from centre 1 and a test set comprised of both an independent cohort of consecutive patients from centre 1 and an external cohort of patients from centre 2 (*n* = 44). Fully-automated MBF was compared between the DS-AIF and AI-AIF methods using the Mann–Whitney U test and Bland–Altman analysis. There was no statistical difference between the MBF quantified with the DS-AIF [2.77 mL/min/g (1.08)] and predicted with the AI-AIF (2.79 mL/min/g (1.08), *P* = 0.33. Bland–Altman analysis shows minimal bias between the DS-AIF and AI-AIF methods for quantitative MBF (bias of −0.11 mL/min/g). Additionally, the MBF diagnosis classification of the AI-AIF matched the DS-AIF in 669/704 (95%) of myocardial segments.

**Conclusion:**

Quantification of stress perfusion CMR is feasible with a single-sequence acquisition and a single contrast injection using an AI-based correction of the AIF.

## Graphical Abstract

**Graphical Abstract:**
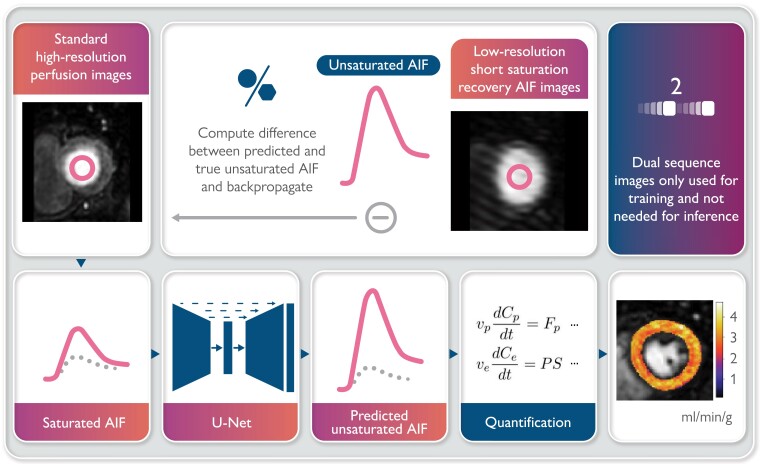

## Introduction

Stress perfusion cardiac magnetic resonance (CMR) is typically performed with a dynamic contrast-enhanced acquisition in which a bolus of a gadolinium-based contrast agent is visualized passing through the left ventricle (LV) and perfusing the myocardium. This is performed under stress conditions using pharmacologically induced vasodilation to assess areas of inducible hypoperfusion. On the weight of evidence from recent clinical trials, stress perfusion CMR is now one of the guideline-backed methods of choice for the identification of myocardial ischaemia.^1^ It has been shown to be highly accurate for the diagnosis of significant coronary artery disease (CAD) and is non-inferior to the invasive reference standard for guiding the management of patients with stable CAD.^2–4^ However, Villa et al.^5^ showed that the diagnostic accuracy of stress perfusion CMR depends on the level of training of the operator, due to the complexity of visually interpreting the scans. Observer-independent quantitative perfusion analysis may overcome this limitation.

The quantification of myocardial blood flow (MBF), by modelling the tracer-kinetics, represents a viable alternative to visual assessment and can be automated.^6^ It, thus, reduces the dependence on the experience of the operator. Additionally, quantitative MBF has been shown to be of independent prognostic value,^7^ allow the detection of coronary microvascular dysfunction,^8^ and provide insight into ischaemia in a range of other cardiovascular conditions.^9–11^ From a technical standpoint, one of the major challenges of MBF quantification is the sampling of the arterial input function (AIF) as required for the tracer-kinetic modelling. The AIF is typically sampled from the basal left ventricular (LV) blood pool or aortic root^12^ and because the whole bolus of contrast passes through the LV cavity more-or-less simultaneously, very high concentrations of contrast agent are recorded at the peak of the AIF. There is known to be a non-linear relationship between the concentration of gadolinium and the measured MR signal, particularly at high concentrations and thus, on the standard acquisitions, the measured signal in the LV is saturated.^13^

There are potential solutions to the AIF saturation but all have challenges for use in routine clinical practice. For example, a dual-bolus injection of contrast agent, one with a lower dose of gadolinium and hence less saturation, can be used but this adds complexity to the scanning.^14^ The dual-bolus increases the potential for errors with the two injections, and has the limitation of measuring the AIF and myocardial tissue curve at different times. Most recent research in quantitative stress perfusion CMR uses a dual-saturation acquisition sequence in which a short saturation time is used to acquire a low-resolution image slice, with reduced signal saturation for AIF estimation, and the myocardium information is subsequently acquired with a standard higher resolution acquisition.^15,16^ As yet, these dual-saturation methods are not available outside of specialized research settings. The increased imaging time required for the extra AIF slice also means that there is less time available to acquire the standard three slices, potentially leading to reduced spatial resolution or image quality, particularly at high heart rates.

**Figure 1:**
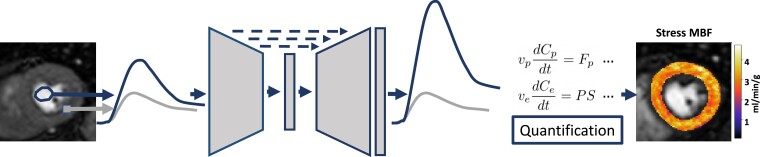
Artificial intelligence-based arterial input function correction: an illustration of the AI-AIF model which takes the saturated AIF from a standard acquisition (left, sampled from the (round) region of interest (ROI) in the LV blood pool) and predicts an unsaturated version which, with the myocardial tissue curve (square ROI), is used in the quantification process to generate a stress MBF map, without the need for any additional input such as from a dual-sequence or dual-bolus acquisition. The neural network model used is a 1-D U-Net model, as is commonly for signal and image processing, which consists of a down-sampling block (encoder) and an up-sampling block (decoder) to reconstruct an output of the same dimensions as the input.

Therefore, there is a clear unmet need for an AIF sampling approach that is both widely available and easy to integrate in clinical routine, ideally with a single-bolus of contrast agent and a commercially available single-saturation sequence acquisition. In this work, we present the artificial intelligence-based AIF (AI-AIF), a deep learning model trained to predict the unsaturated AIF from a saturated single-bolus, single-sequence AIF. This builds on the idea that deep learning models can efficiently learn non-linear mappings, and thus, well-approximate complex physical processes that are otherwise difficult to model, and other recent work on deep learning for parametric mapping,^17^ diffusion modelling,^18^ and tracer-kinetic modelling.^19,20^ Specifically, we show that the non-linear mapping from saturated to unsaturated AIF can be learned from a data set of paired saturated and unsaturated AIFs acquired with a dual-saturation sequence. This deep learning-based non-linear correction can then be applied prospectively to the AIF from standard stress perfusion CMR data to allow accurate quantification without the need for a dual-bolus or dual-sequence acquisition, as shown in *Figure 1*. It is expected that this will increase the availability of quantitative stress perfusion and make it easier to adapt in clinical practice.

## Methods

### Study population

This was a multicentre retrospective study, with data included from two UK centres [King’s College London (centre 1) and the University of Leeds (centre 2)] which was approved by the institutional research ethics committee and complied with the Declaration of Helsinki. All patients included in this study provided written informed consent, in accordance with the National Research Ethics Service approvals (15/NS/0030 and 18/YH/0168, respectively).

The data used in this study consisted of two parts. The training data set was a retrospective sample of patients exclusively from centre 1, and a test set was comprised of both an independent cohort of consecutive patients from centre 1 and an external cohort of patients from centre 2. The external dataset, acquired at a different centre, on a different type of MRI system, and at a different magnetic field strength was included to assess the generalization capacity of the model.

#### Training dataset

The training dataset consisted of a retrospectively collected convenience sample of 201 patients who, between January 2017 and July 2021, underwent cardiac MRI including contrast-enhanced stress perfusion at centre 1 and consented for their anonymized data to be used for research purposes. This dataset was split randomly into 181 and 20 patients for training and model validation, respectively.

#### Test dataset

The centre 1 test dataset retrospectively enrolled 28 consecutive patients scanned between February 2021 and August 2021, and this was supplemented by 16 patients from centre 2 scanned between March 2021 and September 2021.

#### Image acquisition

The CMR examinations were performed using two different types of scanning systems. A 3-Tesla (T) Achieva system (Philips Healthcare, Best, the Netherlands) was used at centre 1 and a 1.5-T Ingenia system (Philips Healthcare, Best, the Netherlands) at centre 2.

The gadolinium-enhanced perfusion studies were performed with a saturation recovery spoiled gradient echo sequence with an optimized dual-sequence AIF slice implementation to allow MBF quantification, as previously described.^15^ The typical imaging parameters were as follows: repetition time 2.2 ms, echo time 1.0 ms, 100 ms, flip angle 15°, and SENSE acceleration factor 1.8. The low-resolution AIF slice was acquired with the same acquisition parameters except for the short saturation recovery time which was 23.5 ms. In addition to the low-resolution AIF slices, three high-resolution short-axis slices were acquired covering the LV. A bolus of 0.075 mmoL/kg Gadobutrol (Gadovist, Bayer AG, Leverkusen, Germany) was injected intravenously at 4 mL/s using an injector pump (Spectris Solaris, Medrad, Bayer AG), followed by 25 mL of saline flush. Stress perfusion imaging was performed during adenosine-induced hyperaemia (140 µg/kg/min for 3 min, increasing to a further 2 min at 175 µg/kg/min and a further 2 min at 210 µg/kg/min if an insufficient stress response had been achieved).

### AI-AIF

The AI-AIF uses a deep learning model that is designed to resolve the issue of signal saturation in the AIF for quantitative stress perfusion CMR. As shown in *Figure 1*, the deep learning model takes the saturated AIF signal from a standard high-resolution stress perfusion scan and predicts the unsaturated AIF signal without the need for any additional input (like the dual-bolus or dual-sequence). The model is trained with data from dual-sequence acquisitions, acquired at an established centre with extensive experience in quantitative stress perfusion CMR. In particular, the unsaturated data from the short saturation acquisition are used to create a reference standard unsaturated AIF and the network was trained to predict this curve from saturated AIF sampled from the standard acquisition. The final AI-AIF model is made available at https://github.com/cianmscannell/ai-aif, along with the code used for model training.

#### Model architecture

A 1D U-Net^21^convolutional network (CNN) was employed which consisted of five resolution steps, with each resolution step being comprised of two 1D convolutional blocks with batch normalization, ReLU activations, and dropout (probability = 0.2). 1D max-pooling and transposed convolutions are used for down- and up-sampling, respectively. This model architecture was chosen empirically based on the validation data.

#### Training details

The model was initialized with He normal weights^22^ and was trained for 20 000 iterations (including data augmentation), with a batch size of 10 using the ADAM optimization algorithm (learning rate, 0.001)^23^ to minimize the mean squared error (MSE) between the predicted and reference standard unsaturated AIFs. The model with the best validation accuracy was chosen.

#### Training data

The training database consists of 1D time curves of paired saturated and unsaturated AIFs extracted from dual sequence acquisitions. The pre-injection portion of the AIF is cropped to begin four beats before the arrival of the contract agent in the LV. The values of both the AIFs are normalized by the maximum of the saturated AIF for both training and inference, and can be later correspondingly unnormalized to allow MBF quantification. The curves are cropped or padded to give 64 time points.

#### Data augmentation

The data augmentation strategy was chosen in order to preserve the quantitative information present in the AIF signal-intensity curves. To achieve this, independent Gaussian noise with zero mean and a standard deviation of 0.02 for the training input and 0.03 for the output was added. This noise was chosen not to substantially impact the signal of the curves but to simulate slightly different realizations of AIF. Furthermore, a random time-offset of 0, 1, 2, or 3 was applied to the starting time of both the input and output to simulate different arrival times of the contrast agent.

### MBF quantification

#### Image analysis

All image analysis and quantification steps are fully-automated. The stress perfusion images were initially corrected for respiratory motion using a previously described motion compensation scheme,^24^ and the segmentation of a ROI for the myocardium was performed using a deep learning-based automated image processing pipeline^6^ that detects the right ventricular (RV) insertion points. The AIF is extracted as the average of pixels over an ROI chosen to comprise the pixels greater than the 75th percentile of intensity values within the subendocardial border of the myocardium segmentation, i.e. in the LV blood pool.^25^

#### Quantification

Since the premise of the AI-AIF and DS-AIF approaches is that they correct for the non-linearity of the MR signal with respect to the concentration of gadolinium, the concentration of gadolinium [*C*(*t*)] can be approximated from the signal intensities (*S*(*t*)) using a relative signal enhancement conversion^26^:

$$\;\;\;\;\;\;\;\;\;\;\;C(t)=\frac{1}{r_{1}\cdot T_{1b}}\left(\frac{S(t)-S(0)}{S_{\mathrm{LV}}(0)}\right)\;\;\;\;\;\;\;\;\;\;\;\;\;\;\;\;\;\;\;\;\;\;\;\;\;\;\;\;\;\;\;\;\;\;\;\;\;\;\;\;\;\;\;\;\;\;\;\;\;\;\;\;\;\;\;\;\;\;\;\;$$

with the *T*_1*b*_ of blood taken as 1736ms at 3-T or 1435 ms at 1.5-T and *r*_1_ the contrast agent as 4.5 s^-1^ mmoL//L ^­^_­_,^27^*S*_LV_ is the signal in the LV blood pool. Quantification of MBF is then undertaken by deconvolving the AIF and myocardial tissue curves on a pixelwise level, using a Fermi function-constrained deconvolution.^28^

### Evaluation

We evaluated the AI-AIF with respect to the reference standard dual-sequence AIF (DS-AIF) in two stages. In the first stage, the signal-intensity curve of the AI-predicted unsaturated AIF is compared with the DS-AIF. Second, the effect of the differences between the predicted and reference DS-AIFs on the downstream task of stress MBF quantification is assessed.

#### AIF curve

The median (interquartile range) (IQR) normalized mean squared error (NMSE) between the AI-predicted and reference DS-AIFs is reported. Additionally, the difference in peak values (PV), time-to-peak (TTP), and full width at half maximum (FWHM) of the AIFs is evaluated, and the distributions of PV, TTP, and FWHM of the AI-AIFs are compared with the distributions of PV, TTP, and FWHM of the DS-AIFs using a Mann–Whitney U test, and the distributions are visualized with a boxplot.

#### MBF quantification

Stress MBF is quantified using both the AI-AIF and DS-AIF for all patients in the test set, and is reported as median (IQR), with a Mann–Whitney U test, used to test for significant differences between the AI-AIF and DS-AIF measurements. Bland–Altman analysis was used to assess the bias and limits of agreement between the manual and automated analysis. The linear relationship between stress MBF derived with AI-AIFs and DS-AIFs is visualized with the equation of the line of best with and the associated *R*^2^ value is also reported. This evaluation is performed both on a patient-wise level (MBF averaged over all pixels from each patient) and on an American Heart Association (AHA) 16 segment-wise level.^29^ To assess the generalization performance of the AI-AIF model to external data, a further Mann–Whitney U test is performed to test for differences, between internal and external testing data, in the difference between AI-AIF and DS-AIF-derived stress MBF. All statistical analysis was performed in Python using SciPy.^30^

In order to also assess the effect of differences in stress MBF between the AI-AIF and DS-AIF methods on the diagnostic accuracy of the quantitative stress MBF values, a further evaluation was conducted with respect to the optimal cut-off threshold for CAD. In particular, a diagnosis would be changed by the use of the AI-AIF if the stress MBF value was lower than the threshold with the AI-AIF and higher with the DS-AIF or vice versa. The diagnosis would be unchanged by the use of the AI-AIF if stress MBF was lower than the threshold with both the AI-AIF and DS-AIF approaches or higher than the threshold with both approaches. In the ideal scenario, the use of the AI-AIF instead of the DS-AIF should leave the diagnosis of all vessels unchanged, and so the percentage of AHA segments for which the diagnoses match is reported. In addition, since the diagnosis for a coronary vessel is made based on the average MBF of the two lowest AHA segments in that coronary territory,^31^ the percentage of vessels for which the diagnoses match is also reported. In this study, the quantitative MBF threshold is taken to be 1.35 mL/min/g, as found to be optimal by Hsu et al.^32^ using similar methods.

## Results

### Study population

The test data set baseline characteristics are summarized in *Table 1*. The AI-AIF model was applied to all patients in the test data set and quantitative perfusion analysis was successfully performed in all 704 AHA segments with both the DS-AIF and AI-AIF methods.

**Table 1:** Baseline characteristics

Characteristic	All (*n* = 44)
Age	63 (17)
Female sex	15 (34%)
Hypertension	17 (39%)
Diabetes mellitus	10 (23%)
Hyperlipidaemia	6 (14%)
(Previous) Smoker	5 (11%)
Prior history of CAD	21 (48%)

A summary of the baseline characteristics of the test set patient cohort. Data are shown as median (IQR) or n (%).

### AIF curve

**Figure 2:**
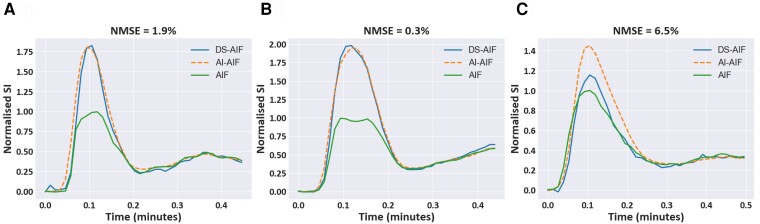
Example comparison of AI-AIF and DS-AIF curves. A representative set of AIF curves from the test set showing both the DS-AIF and AI-AIF curves in comparison to the saturated standard AIF. (*A*) and (*B*) show strong agreement between the DS-AIF and AI-AIF, as evidenced by the low NMSE. (*C*) shows a less strong agreement in a case where the DS-AIF does not markedly correct for saturation in the AIF.

**Figure 3:**
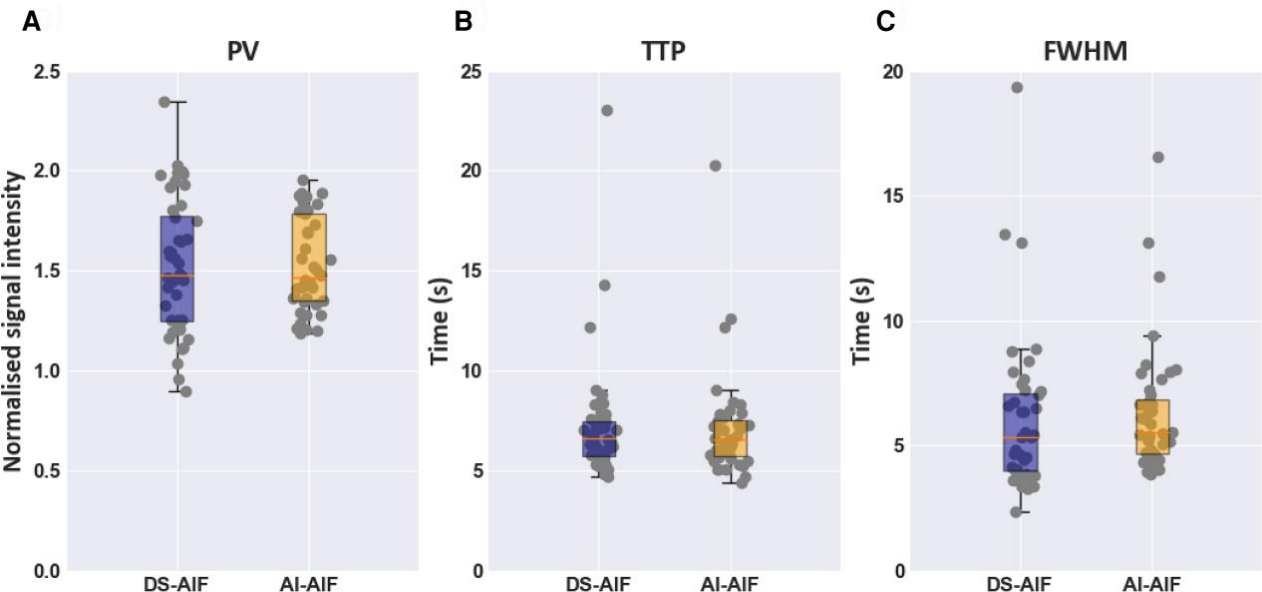
Quantitative comparison of AI-AIF and DS-AIF curves. Boxplots comparing the distributions of values of the quantitative curve metrics PV (*A*), TTP (*B*), and FWHM (*C*) between the DS-AIF and AI-AIF curves. There is no statistically significant difference between any of the pairs of distributions.

*Figure 2* compares the predicted AI-AIF with the reference standard DS-AIF and the standard high-resolution AIF for three representative patients. A strong agreement is shown in *Figure 2A* and *B* with less good agreement seen in *Figure 2C*. The median NMSE between the DS-AIF and AI-AIF curves was 1.9% (2.5). While the average agreement is good, a similarly bad agreement to *Figure 2C* (NMSE ≥ 6.5%) is found in 5/44 (11.4%) of test cases. In addition to being similar in terms of absolute error, the quantitative metrics which describes the curves (PV, TTP, and FWHM) are similar between the DS-AIF and AI-AIF, as shown in *Figure 3*. There were no significant differences in any of these metrics between the DS-AIF and AI-AIF. The median PV (in normalized signal-intensity units) was 1.48 (0.53) for the DS-AIF and 1.47 (0.44) for the AI-AIF (*P* = 0.94), the median TTP was 6.60 s (1.75) for the DS-AIF and 6.60 s (1.76) for the AI-AIF (*P* = 0.99), and the median FWHM was 5.34 s (3.09) for the DS-AIF and 5.54 s (2.18) for the AI-AIF (*P* = 0.21). This indicates that the AI-AIF model can correct for the signal saturation in a standard single-sequence acquisition and yield curves that closely match those acquired with a dual-sequence.

### MBF quantification

**Figure 4:**
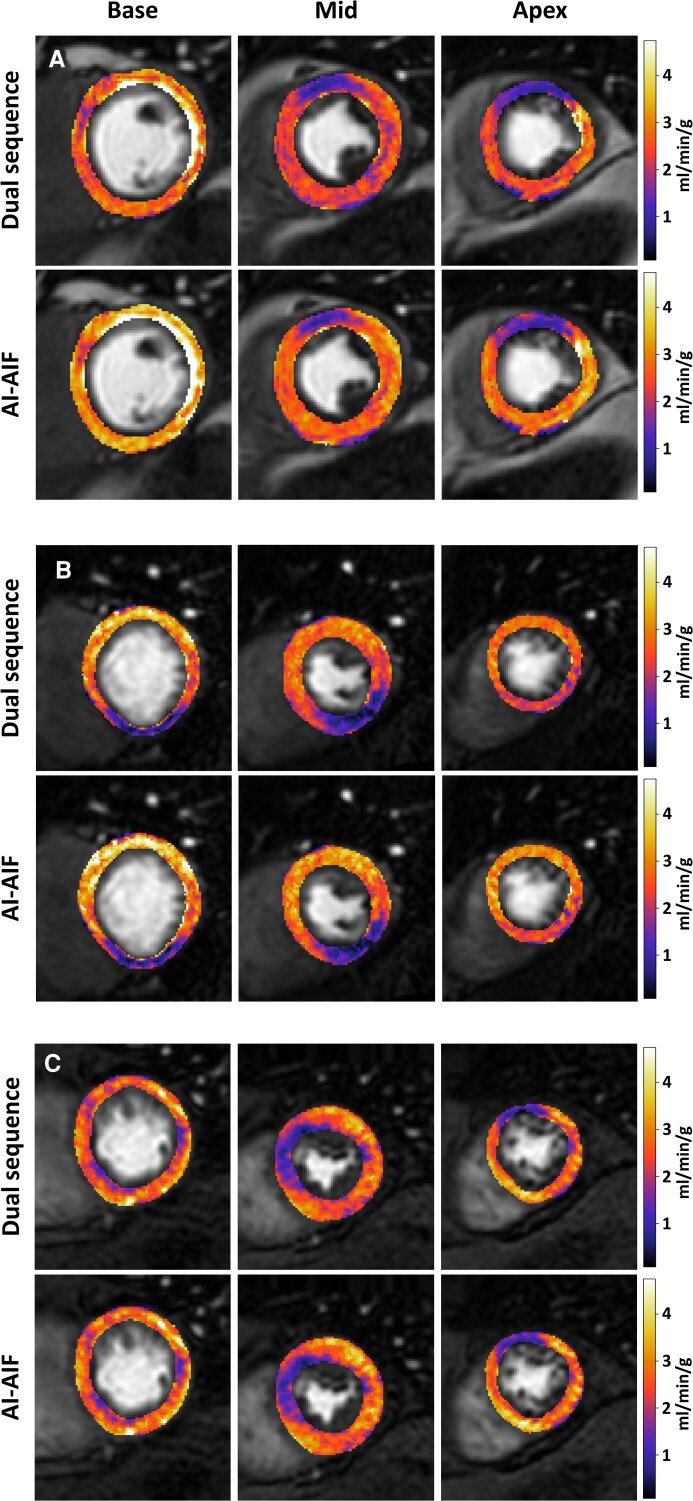
Quantitative MBF maps. A comparison of quantitative MBF maps with both the DS-AIF and AI-AIF for a representative sample of patients, (*A*) and (*B*) from centre 1 and (*C*) from centre 2. There is seen to be a close agreement between the methods, and despite subtle differences, the diagnostic information is visually preserved with the use of the AI-AIF.

The median MBF was 2.77 mL/min/g (1.08) quantified with the DS-AIF and 2.79 mL/min/g (1.08) quantified with the AI-AIF. There was no statistically significant difference between the approaches (*P* = 0.33). There were also no significant differences between the median MBF for the subgroup of patients from centre 1 (2.39 mL/min/g (1.02) vs. 2.49 mL/min/g (1.24), *P* = 0.31) or the subgroup of patients from centre 2 (3.01 mL/min/g (0.65) vs. 3.08 mL/min/g (1.08), *P* = 0.49). Three example patients comparing pixelwise MBF maps between the DS-AIF and AI-AIF are shown in *Figure 4*. Though subtle differences are apparent between the DS-AIF and AI-AIF-derived maps, the diagnostic information appears to be preserved. There was a significant difference between median MBF at centre 1 of 2.39 mL/min/g (1.02) vs. at centre 2 of 3.01 mL/min/g (0.65) with the DS-AIF, and 2.49 mL/min/g (1.24) vs. 3.08 mL/min/g (1.08) with the AI-AIF (*P* < 0.01 and *P* = 0.02, respectively). However, there was no significant difference (*P* = 0.11) in the median difference in MBF between the DS-AIF and AI-AIF at centre 1 [−0.23 mL/min/g (0.48)] and centre 2 [−0.11 mL/min/g (0.62)]. This indicates that the AI-AIF performs similarly well at both centres and that the difference in MBF values between centre 1 and centre 2 was a result of the different patient cohorts or differences in the imaging system related to the field strength or the pulse sequence.

**Figure 5:**
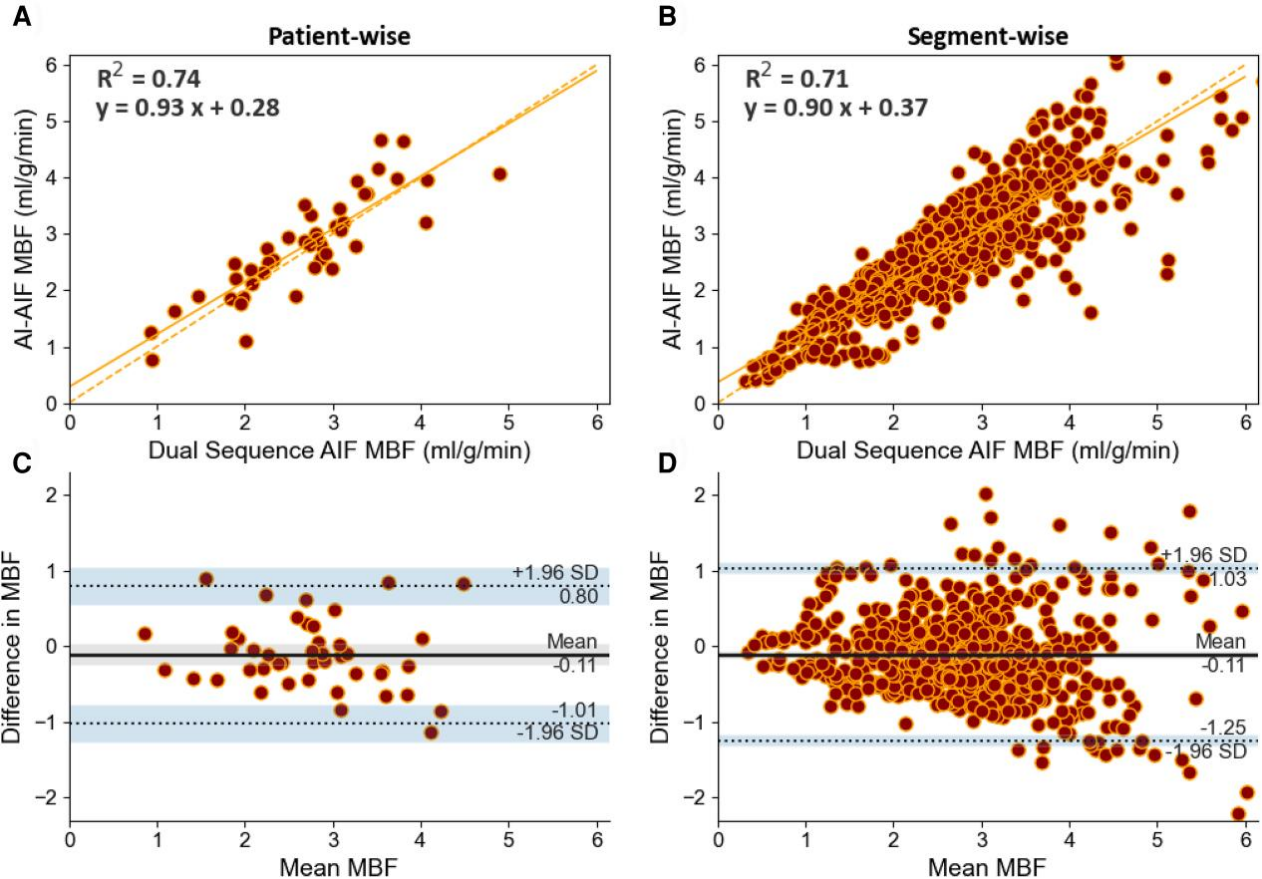
Comparison of overall MBF values between the DS-AIF and AI-AIF approaches. This shows scatter plots of MBF with the DS-AIF vs. MBF with the AI-AIF, with the line of best fit, its equation and associated $R^{2}$ values (*A*) and (*B*), and the Bland–Altman analysis (*C*) and (*D*) on a per-patient (left column) and per-AHA segment basis (right column). The shaded regions represent the 95% confidence intervals for the bias and limits of agreement.

There was a strong linear relationship between the MBF values estimated with the DS-AIF and AI-AIF approaches on both per-patient (*y* = 0.93*x* + 0.28 with the *R*^2^ value of fitting 0.74, *Figure 5A*) and per-segment (*y* = 0.90*x* + 0.37 with the *R*^2^ value of fitting 0.741, *Figure 5B*) levels. Additionally, the Bland–Altman analysis (per-patient in *Figure 5C* and per-segment in *Figure 5D*) shows minimal bias between the DS-AIF and AI-AIF methods for quantitative MBF with a mean bias of −0.11 mL/min/g and limits of agreement that are in line with the inter-study repeatability of stress MBF values.^33^ Finally, the effect on the diagnostic accuracy of the use of the AI-AIF in place of the DS-AIF was assessed. The classification of CAD with respect to the optimal MBF threshold agreed for 95.0% (669 out of 704) AHA segments and 89.4% (118 out of 132) coronary vessels.

## Discussion

In this work, a deep learning-based correction of the signal saturation in the AIF for quantitative stress perfusion CMR is proposed which has the potential to solve the long-standing issue of the AIF estimation for accurate quantification. To date, there has been limited evidence of quantitative stress perfusion CMR without the use of a modified acquisition scheme, either a dual-bolus contrast injection scheme or a dual-saturation acquisition sequence, but there has been no clinical validation or adoption.^34^ The need for modified acquisitions has limited the use of quantitative stress perfusion CMR thus far, but the AI-AIF model has been shown to allow the quantification of perfusion CMR with both a single contrast injection and a standard single-saturation acquisition sequence. Crucially, this is simpler and more available than any other approach for MBF quantification.

Even though the dual-bolus protocol can be used without any additional technology, its clinical adoption has been limited due to the complexity added to the scan and the extra work involved in the two injections. Also, despite being proposed nearly 20 years ago,^35^ there has been no widely available implementation of the dual-sequence and no commercial solution, so it is limited to use in a small number of research centres. The code for the AI-AIF model developed in this work is provided as open-source, and thus, is widely available, making it easy to use and not dependent on the availability of technology or the local experience. It is also easily compatible with existing software for quantifying MBF,^6^ and so, it can be used in a fully-automated manner. The relative simplicity of the method and availability of the code also makes it straightforward to integrate the AI-AIF with existing perfusion quantification software.

The AI-AIF will not only simplify the scanning workflow, which would make the imaging more likely to be performed and more available, but it is also less prone to human error. The image analysis and post-processing are further simplified, especially compared with the dual-sequence in which an additional imaging slice is acquired that then needs customized processing methods for image registration, segmentation, and proton-density correction. The AI-AIF only requires the standard high-resolution slices, and thus, no customized image processing. As well as being an important future step towards clinical adoption and standardization, since the AI-AIF model is a post-processing step, it will facilitate the retrospective analysis of data acquired without a dual-bolus or dual-sequence. An important application of this would be to retrospectively quantify data from clinical trials, which used visual assessment only, to build further evidence for the use of quantitative MBF.

The results presented in this study evaluate the AI-AIF model compared to the reference standard DS-AIF considering both the direct correspondence of the AIF curves, the quantitative MBF values derived with the AIFs, and the effect of differences in the quantitative MBF values on the patient’s diagnosis. This analysis used both an independent cohort of patients from centre 1 and an external cohort of patients from a second centre to give an idea of the real-world performance with data acquired at a different centre with a different imaging system and a different magnetic field strength. These results are considered to be promising, but it is acknowledged that further clinical validation is required before the potential adoption of the AI-AIF.

As well as closely matching in terms of NMSE (<2%), the AIF curves from the AI-AIF were not significantly different in terms of PV, TTP, or FWHM. The close match in curves resulted in no significant difference in quantitative MBF between the AI-AIF and DS-AIF approaches. The Bland–Altman analysis showed minimal bias and 95% limits of agreement that were in line with the inter-study repeatability of stress MBF values^33^ on both per-segment and per-patient levels. Higher MBF was found in the external data from centre 2 but this may be because of differences in the data acquisition or patient cohorts, and even in this subgroup, there is no significant difference between the AI-AIF and DS-AIF. There was no increase in the difference between AI-AIF-based and DS-AIF-based MBF values between the internal and external testing data suggesting that the model can generalize well to new data. It was further shown that, according to a previously published diagnostic cut-off of 1.35 mL/min/g, the diagnosis of the AI-AIF matched the DS-AIF in 669/704 (95%) of segments, indicating that the AI-AIF model can be used without sacrificing diagnostic accuracy compared with the DS-AIF. While these results are generally positive, there were isolated cases with less good agreement for which the resulting analysis may warrant closer inspection if they are to be used for clinical decision-making.

The implementation of the AI-AIF as a retrospective correction step on the AIF curve maintains its flexibility to be used with data from different types of pulse sequences and acquisition schemes. For example, recent work has investigated the acquisition of additional slices to increase spatial coverage^36^ but these adaptations would not affect the applicability of the AI-AIF as it can be applied to the AIF curves regardless of how they have been acquired. It is also straightforward to use—so it will not be limited to use in experienced centres with advanced research programs. Since, in addition to the trained model, the scripts for training are also provided, it could also be retrained to adapt to different types of data and improve robustness. This is also relevant for other imaging modalities as it is a general solution, and as discussed by Murthy et al.^37^ saturation also occurs with high doses of injected radiotracer activity for perfusion imaging on contemporary 3-dimensional PET systems.

This work focussed on stress MBF only, as opposed to both stress and rest MBF, as it has been shown that the inclusion of the rest images does not improve the diagnostic accuracy.^32^ This is in line with recent research^38^ and clinical guidelines^39^ which suggest the omission of the rest images in order to reduce the scan time. However, the AI-AIF could be easily extended by including the rest images in the training data. Further limitations include that despite testing the model on data from different centres and magnetic field strengths, the data used were from a single scanner manufacturer. Retraining with new data would likely be required to transfer the solution to work with data from other scanner manufacturers. As discussed, a wider variety of patient data sets could also be added to the model training in future work to improve robustness and mitigate potential failures of the model, as shown in *Figure 2C*. Here, there is a large difference between the AI-AIF and DS-AIF, and this case is shown to represent the worst case in the test set. However, there is seen to be no correction of the AIF with the dual sequence, which is questionable, and shows the limitation of the lack of a true gold standard for validation. The dual-saturation reference standard itself is designed to minimize saturation rather than eliminating it entirely. There may still be residual saturation, for example, due to T2* effects, but it has previously been shown that these effects are small.^40^ Furthermore, the quantification step used the simplistic Fermi function deconvolution, and future work could extend this to more complex quantification models,^41^ which could even be done using deep learning in combination with the AI-AIF in one model.

This study represents a promising initial proof of concept for applying AI to correct the signal saturation in the AIF for quantitative stress perfusion CMR. However, further work will be required to enable widespread clinical adoption of the approach. In this study, the AI-AIF was compared to the reference standard DS-AIF but the quantitative MBF values obtained with the AI-AIF would need to be validated vs. fractional flow reserve, which is considered the gold standard for identifying ischaemia-related stenosis.

Although the presented test results indicate the performance level in a representative and challenging cohort of patients (widespread cardiovascular risk factors and 48% with a prior history of CAD), the studied cohort is still relatively small. The inclusion of more patients for training will help the capacity of the model to deal with less common cases and more patients for testing will improve confidence in the performance. A more extensive use of data augmentation could also achieve more variability in the training data and a possibility to realize this would be to use a (deep) generative model to generate AIFs for training. Further improvements to the model could be possible by exploring new model architectures or physics-informed learning schemes, but these were not studied in this work, and the U-Net is currently considered one of the methods of choice for signal and image processing.

The availability of the AI-AIF leading to more simplified acquisitions combined with the extensive validation,^31,32,42^ established prognostic significance,^7,43^ and the advantages of CMR perfusion over single photon emission computed tomography (SPECT) or positron emission tomography (PET) perfusion, including the superior spatial resolution and lack of ionizing radiation, may finally pave the way for more widespread clinical adoption of stress perfusion CMR. As discussed, stress perfusion CMR is gaining clinical relevance due to the growing body of evidence from randomized controlled trials supporting its use^2–4,44^ but the quantification of MBF will help to reduce the operator-dependence,^5^ highlighting the need for accessible quantitative methods. Even more significantly, the quantification of MBF has now been recommended in the American guidelines for the evaluation of patients with chest pain^1^ as it is crucial for the evaluation of patients with ischaemia and non-obstructive CAD.^8,42,45^ The proposed method will be important in this context as it makes quantifying stress perfusion CMR as easy as quantifying alternative functional perfusion tests, such as PET. With similar ease-of-use, quantitative perfusion CMR may become the method of choice, due to its higher spatial resolution, as subendocardial ischaemia is a key feature in these patients.^46,47^

## Conclusion

This study presents a step towards the widespread availability of quantitative stress perfusion CMR, an approach for accurate quantification of stress perfusion CMR from single-bolus and single-saturation sequence scans, without any modified acquisition. It uses a deep learning model to correct the signal saturation in the AIF which has been trained using an extensive database of unsaturated AIFs acquired with a dual-saturation acquisition sequence. This is an important step in alleviating the need for labour and time-intensive dual-bolus protocols and for proprietary dual sequence acquisitions, for which there are variable levels of availability. The approach is easy to reproduce or extend as the training code is available, it does not add processing time, and uses a simple model that could be directly incorporated with the scanner. The AI-AIF has the potential to finally advance quantitative stress perfusion CMR from the research domain to integration in routine clinical care.

## Data Availability

The derived data and codes to reproduce the results of this study are available at https://github.com/cianmscannell/ai-aif. The original patient data may be made available upon request to the corresponding author.

## Lead author biography

Dr Cian M. Scannell studied Mathematical Sciences at University College Cork (Ireland) where he graduated with first-class honours in 2016. He then moved to King's College London to undertake his master's and PhD degrees as part of the Centre for Doctoral Training in Medical Imaging. Here, he worked on a thesis entitled “Automated quantitative analysis of first-pass myocardial perfusion magnetic resonance imaging data”. In 2021, he received his PhD and then continued for a further year as a postdoctoral researcher funded by the Wellcome/EPRSC Centre for Medical Engineering at King's College London with a project on the development of robust and generalisable models for clinical integration. In 2022, he was appointed assistant professor with the Medical Image Analysis group in the Department of Biomedical Engineering at the Eindhoven University of Technology. Mandatory photo credit: Vincent van den Hoogen.
